# Dynamic Epidemiological Models for Dengue Transmission: A Systematic Review of Structural Approaches

**DOI:** 10.1371/journal.pone.0049085

**Published:** 2012-11-06

**Authors:** Mathieu Andraud, Niel Hens, Christiaan Marais, Philippe Beutels

**Affiliations:** 1 Centre for Health Economics Research and Modelling of Infectious Diseases (CHERMID), Vaccine & Infectious Disease Institute (VAXINFECTIO), University of Antwerp, Antwerpen, Belgium; 2 Interuniversity Institute of Biostatistics and Statistical Bioinformatics, Hasselt University, Diepenbeek, Belgium; 3 School of Public Health and Community Medicine, University of New South Wales, Sydney, NSW, Australia; University of Hong Kong, Hong Kong

## Abstract

Dengue is a vector-borne disease recognized as the major arbovirose with four immunologically distant dengue serotypes coexisting in many endemic areas. Several mathematical models have been developed to understand the transmission dynamics of dengue, including the role of cross-reactive antibodies for the four different dengue serotypes. We aimed to review deterministic models of dengue transmission, in order to summarize the evolution of insights for, and provided by, such models, and to identify important characteristics for future model development. We identified relevant publications using PubMed and ISI Web of Knowledge, focusing on mathematical deterministic models of dengue transmission. Model assumptions were systematically extracted from each reviewed model structure, and were linked with their underlying epidemiological concepts. After defining common terms in vector-borne disease modelling, we generally categorised fourty-two published models of interest into single serotype and multiserotype models. The multi-serotype models assumed either vector-host or direct host-to-host transmission (ignoring the vector component). For each approach, we discussed the underlying structural and parameter assumptions, threshold behaviour and the projected impact of interventions. In view of the expected availability of dengue vaccines, modelling approaches will increasingly focus on the effectiveness and cost-effectiveness of vaccination options. For this purpose, the level of representation of the vector and host populations seems pivotal. Since vector-host transmission models would be required for projections of combined vaccination and vector control interventions, we advocate their use as most relevant to advice health policy in the future. The limited understanding of the factors which influence dengue transmission as well as limited data availability remain important concerns when applying dengue models to real-world decision problems.

## Introduction

Dengue is a vector-borne disease recognized as the major arbovirose (arthropod-borne virus) in the world with more than 50 million dengue fever cases per year [1], [2]. The major vector, *Aedes aegypti*, thrives in tropical regions, mainly in urban areas, closely linked to human populations providing artificial water-holding containers as breeding sites [3], [4]. A second potential vector, *Aedes Albopictus*, resides in temperate regions (North America and Europe), where it may give rise to occasional dengue outbreaks [5], [6], [7], [8].

Four immunologically distant dengue serotypes (DEN-1, DEN-2, DEN-3 and DEN-4) coexist in many endemic areas [9], [10]. Infection with one serotype has been shown to provide life-long immunity to that serotype but no or only short-term immunity to the other serotypes [9], [10], [11]. The clinical features of dengue have a broad spectrum: most infections remain asymptomatic or induce flu-like symptoms (dengue fever (DF)). Dengue Hemorrhagic Fever (DHF) and Dengue Shock Syndrome (DSS) are the most severe expressions, with case-fatality ratios (CFR) varying from less than 1% to 13% depending on regions and hospitals [12], [13], [14]. In previously infected persons, subsequent infection with another dengue serotype leads to clinical disease for most serotype combinations, and is considered a major risk-factor for DHF/DSS [1], [9], [15].

Johansson *et al.* published a review of mathematical approaches to study dengue transmission dynamics with a focus on estimation methods for the basic reproduction number and their consequences for the impact of vaccination [16]. The present paper reviews research articles of deterministic mathematical models of dengue transmission in humans. Although a large part of the models we review was also briefly discussed in Johansson et al [16], we present a more detailed assessment of model structures, and link this with the underlying assumptions based on epidemiological and entomological studies. These model structures are explored and discussed regarding their influence on projections of the potential impact of vector-control and/or vaccination options.

## Methods

### Search Strategy

We performed a literature search in standard databases (PubMed and ISI Web of Knowledge) up to March 2012. In each database, the keywords “Dengue Epidemic Model” and “Dengue Epidemiological Model” were systematically used. Moreover, since models involving multiple strains may not be specifically developed for dengue infection but would be well equipped to study this problem as a direct application, the stand alone search term, “Multistrain”, was also used. Both MeSH and free-text terms were included in the search procedure, resulting in a preselection of 655 peer-reviewed articles (including duplicates, Table 1). Sixteen articles were excluded (8 Spanish, 5 Portuguese and 3 French) because of non-English language.

**Table 1:** Literature Search Strategy.

Search terms	PubMed	ISI Web Of Knowledge	Total (duplicates)
Dengue epidemic model	84	129	213 (53)
Dengue epidemiological model	173	57	230 (29)
Multistrain	90	122	212 (79)
Total (duplicates)	347 (74)	308 (31)	655 (266)

### Selection

Titles and abstracts resulting from the search described above were screened and research articles on virology and/or immunology were discarded. Articles were included for review if they met the following criteria:

1. representation of the dengue infection process at the host level (excluding studies focusing on entomological aspects only).
2. deterministic approaches using systems of ordinary differential equations (ODE).

An additional implicit selection criterion of focusing the review on unique model structures is that we exclude papers, which use a previously described model structure to estimate reproduction numbers and/or epidemiological parameters from field data.

We refer to references [16] and [17] for reviews specifically dedicated to estimation methods of the basic reproduction numbers from field data. Both stochastic and spatial models were excluded since non-spatial deterministic approaches provide a good mean-field approximation of the system behaviour and preserve the time series pattern of infected hosts, even while ignoring the stochastic features of the dynamics. However, all these excluded approaches (spatial, stochastic and parameter estimations) are briefly discussed in the final section of the paper.

**Figure 1:**
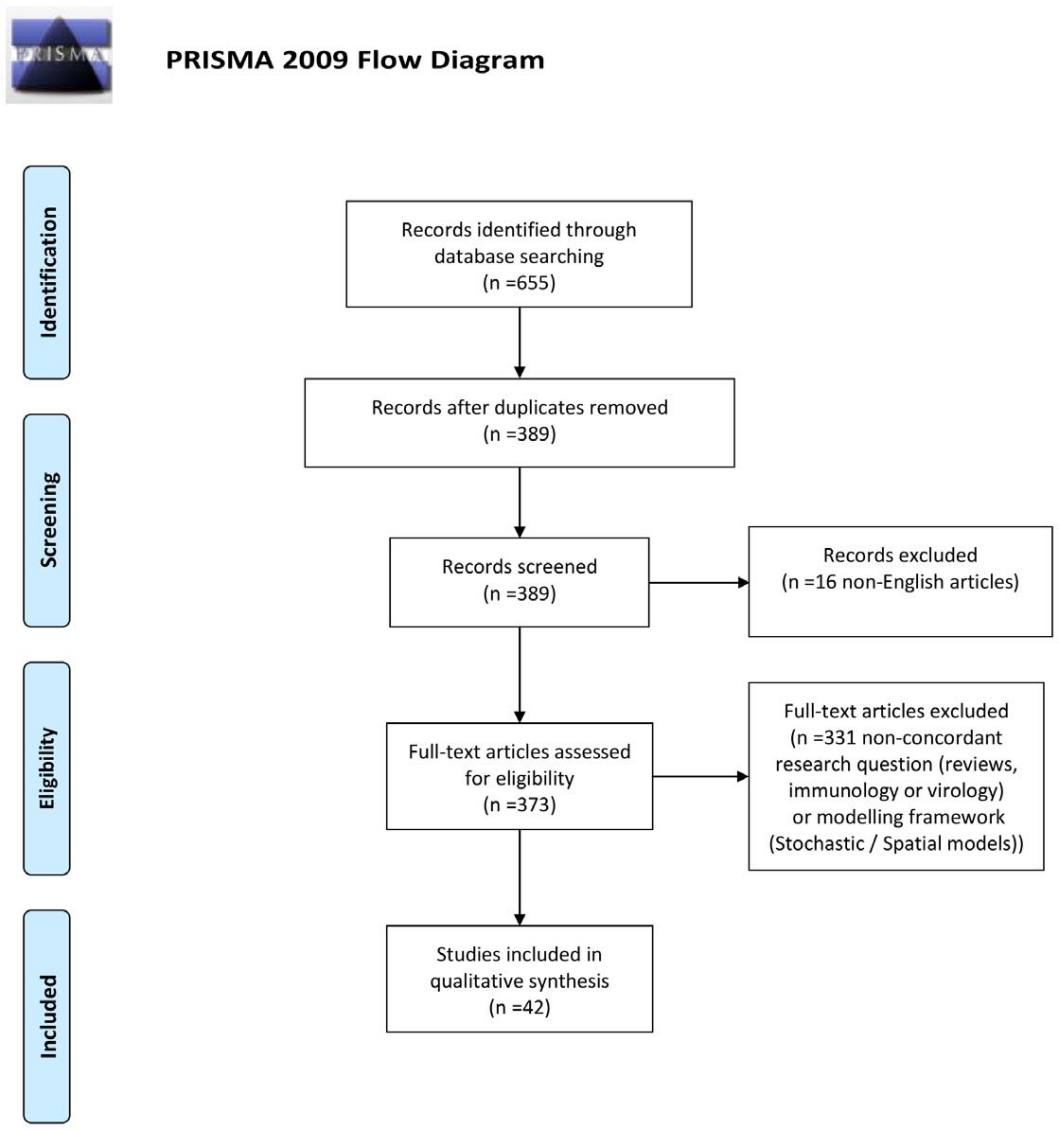
Flow chart representation of the selection process. Sixteen were excluded because of non-English language: Spanish (8), Portuguese (5) and French (3) in the first step of the selection process.

The search and selection process is displayed in Figure 1.

## Results

### Flow of included studies

**Figure 2:**
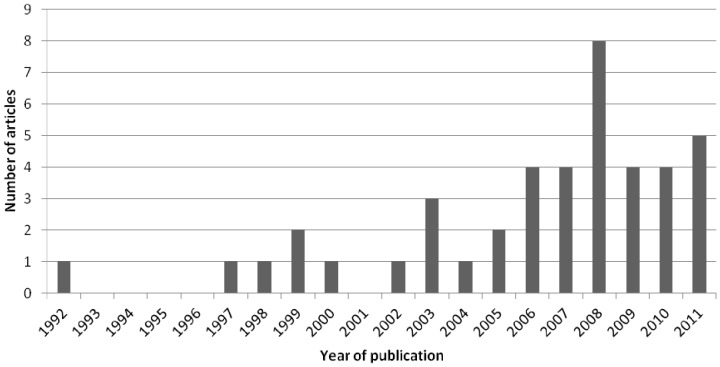
Distribution of the number of articles according to the year of publication.

The number of published dengue models increased drastically during the last two decades (Figure 2). Among the 373 preselected articles (excluding 266 duplicates and 16 non-English articles), 42 models met the selection criteria. Halve of these models were published in the last four years (Figure 2). These models were developed to understand the dynamics of infection and to evaluate the effectiveness and/or cost-effectiveness of control strategies.

### Vector-borne transmission model terminology

The basic (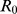) and the effective (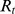) reproduction numbers are defined as the average number of infections produced by a typical infectious individual during his/her entire infectious period in a population that is at time 0 completely (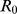) and at time t partially (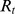) susceptible, respectively. These general key parameters determine whether an infection can invade (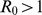) and persist (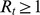) in a population. However, in the case of a vector-borne disease, the time period over which 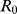 is defined covers a complete vector-host cycle and the involvement of the vector population leads to the use of specific terminology:

Recruitment rate: Since only adult female mosquitoes are involved in the transmission process, most models only represent the adult stage of (female) mosquitoes, ignoring the previous aquatic stages (eggs, larvae and pupae). The recruitment rate corresponds to the inflow of vectors (*i.e.* adult females) in the system. Most studies consider a constant recruitment rate, assuming the maturation of a fraction of a large amount of eggs, independently of the adult population size.

Oviposition rate: Some models represent both aquatic (pre-adult) and winged (adult) stages of vector development. The oviposition rate is the mean number of eggs laid per female per time-unit.

Maturation rates: The mosquito life cycle includes three aquatic (egg, larva and pupa) and one adult (winged) stages. Maturation rates correspond to the inverse of the average duration spent in the different aquatic stages.

Biting rate: average number of bites per mosquito per time-unit.

Extrinsic Incubation Period (EIP): time-interval between a mosquito's infection and when its bites become infectious (latency). Correspondingly, the latent period in hosts is called the Intrinsic Incubation Period (IIP).

Vertical transmission efficiency: percentage of eggs vertically infected when laid by one infectious female mosquito.

### Model descriptions

**Figure 3:**
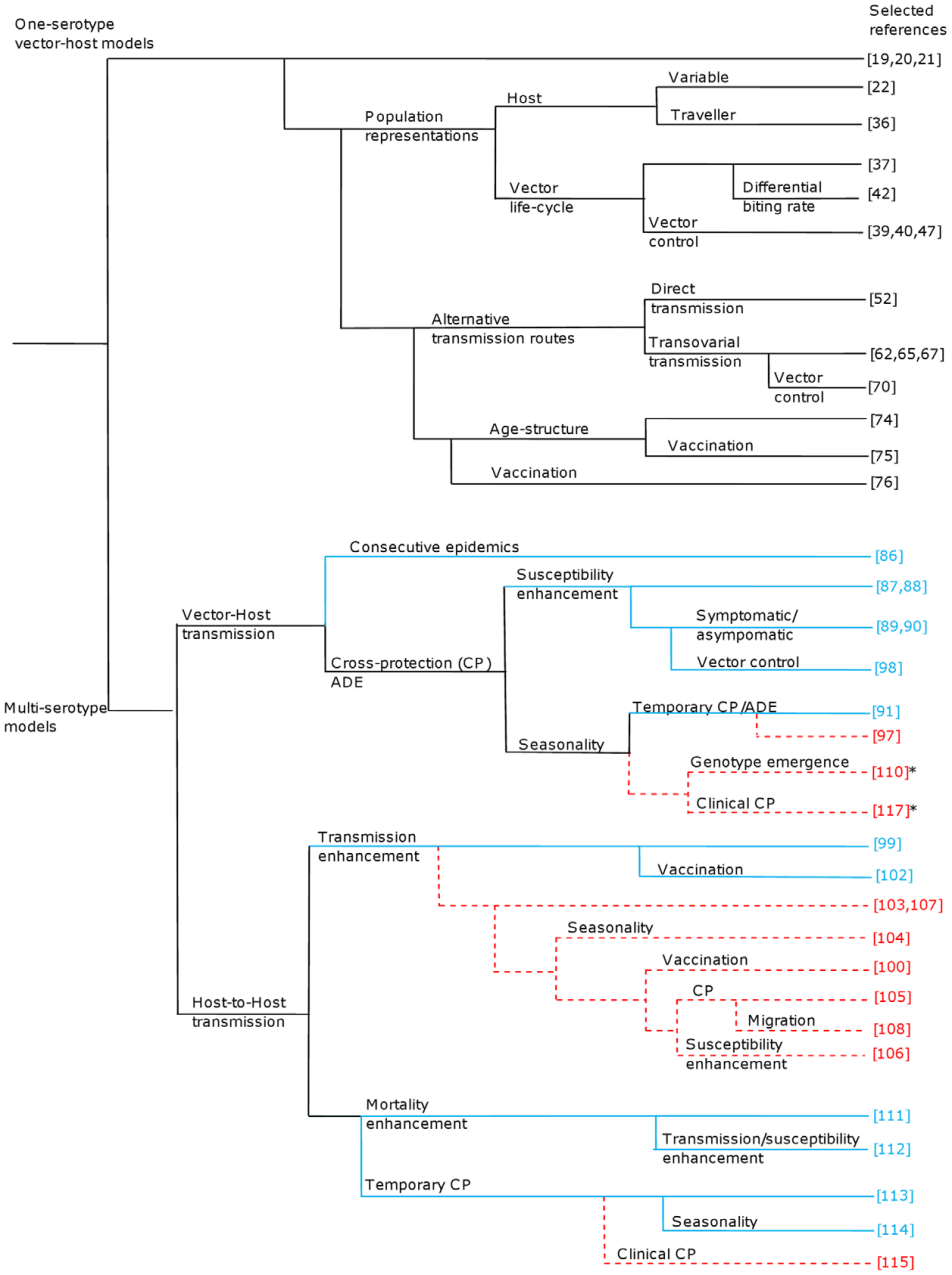
‘Phylogenetic tree’ of selected articles. Models are decomposed according to the number of serotypes considered (one (black lines), two (blue full lines) or more than two (red dashed lines) serotypes. Each branch of the tree corresponds to a modification of the initial model owing to additional assumptions. The word “enhancement” refers to the different modelling assumptions to represent the effect of antibody-dependent enhancement (ADE) and CP stands for Cross-Protection. * Extensions of Host-to-Host transmission models [106], [115] including the vector population.

A “phylogenetic tree”, representing the relationship between the selected articles and the main assumptions for each article, is displayed in Figure 3. This tree has two main branches corresponding to single- and multi-serotype models. Each node reflects the main epidemiological and/or entomological characteristics of the models. Eighteen single-serotype models were based on the vector-host interaction approach with differing assumptions regarding population representation, transmission routes, age-structure, and/or vaccination.

Multi-serotype models were split into two categories:

1. Vector-host transmission (10 articles).
2. Host-to-host transmission (14 articles).

Model structures and underlying assumptions are discussed in the next subsections.

### Single-serotype models

The simple vector-host transmission model described by Bailey in 1975 [18] provides the basis for dengue models addressing a single serotype. The host population was represented by an SIR model, whereas, once infected, the vector-mosquito was assumed to remain infectious until death (SI model):
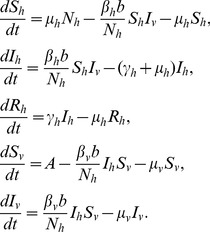
(1)Here, 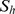, 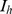 and 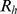 represent the numbers of susceptible, infectious and immune (recovered) hosts; 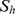, 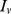 the numbers of susceptible and infectious mosquitoes. 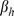 and 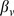 are the vector-host and host-vector probabilities of transmission, respectively; 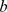 is the biting rate. 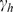 and 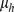 represent the recovery and mortality rates for hosts and 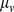 the vector mortality rate. The vector recruitment rate 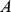 was assumed to be constant. Parameter definitions and the range of values used in the various models are displayed in Table 2.

**Table 2:** Definitions and ranges of the main parameters in vector-host transmission models.

Parameter	Definition	Value
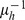 (year)	host life expectancy = (host recruitment rate)^−1^	50–70
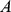 (day^−1^)	vector recruitment rate	400–5000*
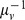 (day)	vector life expectancy	4–50
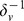 (day)	extrinsic incubation period	8–12
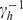 (day)	infectious period	3–14
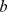 (day^−1^)	biting rate	0·3–1
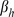	probability of transmission from vector to host	0·1–0·75
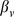	probability of transmission from host to vector	0·5–1

* The range for the vector recruitment rate was derived from modelling studies considering exclusively the adult mosquito population with a constant recruitment rate (*i.e.* a constant vector population) and providing parameters values for numerical simulations [19], [21], [76], [88].

Esteva and Vargas derived from model (1) the threshold value governing the stability of disease free and endemic equilibria [19]. As Esteva and Vargas, Tewa *et al.* concluded that the disease-free equilibrium is stable whenever 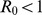, the endemic equilibrium being globally stable otherwise [20]. This model was used to study the effectiveness of ultra-low volume (ULV) insecticide dispersion: after a momentary decrease in the vector population, the vector density reverts back to the pre-treatment level, only inducing a delay in the epidemic curve. These results, in line with a previous study [21], are a direct consequence of the constant recruitment rate, leading to an asymptotic vector population (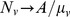 as 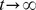), and global stability of the endemic equilibrium whenever 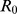 is greater than 1.

A large range of models were derived from model (1), reflecting different assumptions associated with population representations, dengue epidemiology, and/or the transmission routes.

#### Population representations

Esteva and Vargas extended their previous model assuming an exponential growth of the host population and a disease induced mortality [19], [22]. Three threshold parameters, governing the system's behavior, were identified: 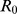 conditional on the existence and stability of an endemic equilibrium, 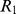 related to the asymptotic behavior of the number of infected humans and 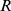 controlling the growth of the human population. Since severe dengue cases result mostly from re-infection with different serotypes, disease-induced mortality in this single-serotype model was not epidemiologically relevant [23], [24].

Dengue infection is also a health issue for travellers in endemic areas, even if severe clinical cases remain relatively rare [25], [26], [27]. The introduction of an expansive laboratory-based notification system allowed to identify 3.85 times more dengue cases in 2002 (231) than in 2001 (60) among German travellers [28], [29]. Travellers can also introduce new serotypes in endemic areas, or dengue virus in non-endemic areas infested by vector-mosquitoes, and play an important role in dengue spread [30], [31], [32], [33], [34], [35]. Pongsumpun *et al.* developed a model including a traveller subpopulation within an endemic area to study the relationship between the length of stay in an endemic area and the proportion of infected travellers [36]. They showed that the risk of infection in travelers was positively correlated with the length of stay, which is consistent with a risk factor analysis for dengue infection in travellers [29]. However, for long periods of migration the proportion of infected travelers approached an asymptote due to the assumption of homogeneous mixing of local and long-term traveler populations.

Erickson *et al.* described the vector population including the pre-adult stages and dividing the adult population in three subgroups: immature, gestating and reproducing adults [37], [38]. They also studied the impact of temperature variations on maturation rates. Dengue virus was introduced through the arrival of infectious mosquitoes at different time periods. Due to temperature-dependence in parameters governing the vector population, the arrival date of the infectious mosquitoes was found essential for the dengue transmission: under unfavorable conditions (low-temperatures) the vector population was too small to sustain dengue outbreaks. Moreover, the authors showed that dengue overwintering was unlikely to occur in temperate areas in absence of transovarial transmission, which primarily occurs in tropical and subtropical regions.

Yang and Ferreira extended the basic model (1) by testing different vector-control strategies (insecticide or larvicide application, removal of breeding containers) [39]. Their model accounted for mosquito maturation stages (eggs, larvae, pupae, adults), and thus relaxed the assumption of a constant recruitment rate. To evaluate the impact of control measures, the authors introduced an efficiency index, defined as the reduction factor of the adult vector population after vector control. This index was then transposed to the host population to evaluate the impact on dengue transmission. Although all control policies were efficient to reduce vector population size, with efficiency index up to 80%, this trend was not observed in the host population in which reduction of dengue cases was estimated below 40%. Luz *et al.'s* [40] vector-host dengue transmission model was part of an economic evaluation of different vector control strategies. They adapted a previous model describing the vector population (egg, larvae; pupae and adult stages), close to Erickson's model [37], accounting for insecticide resistance with fitness cost (increased mortality rate for resistant mosquitoes). Resistant mosquitoes were assumed to remain unaffected by control measures [41]. Although the serotypes were not explicitly identified in the model, one host could incur two successive infections. A period of complete cross-immunity (4 months) was also considered after a first infection, followed by a decrease of susceptibility to a secondary infection. Forty-three combinations of multiple larvicide and adulticide applications were tested, assuming increased mortality rates for targeted vector sub-populations during the period of insecticide activity in the environment. An economic assessment of the different control strategies was made by estimating the reduction in disease burden under each strategy. Yearly larvicide application was shown to significantly reduce the vector population in the short term, yielding moderate health gains (expressed as Disability Adjusted Life-Years (DALY)), in the first two years of vector control. Moreover, the evolution of insecticide resistance combined with a loss of herd immunity, due to lower transmission in the first years, could lead to counterproductive effects increasing the magnitude of potential dengue outbreaks. Alternatively, the most cost-effective strategy consisted of six high efficacy adulticide applications per year, reducing the disease burden to the greatest extent and meeting WHO's standard for a cost-effective intervention. Luz *et al.* used a simplified formulation of their model [40] to investigate the potential impact of increased biting rates in dengue infected mosquitoes [42]. The effect of dengue infection on mosquito feeding behavior is not clearly established and different studies yielded controversial results [43], [44]. However, two recent experimental studies support the differential biting rate assumption [45], [46]. Using numerical simulations, Luz *et al.* showed that an increase of 50% of the biting frequency would produce an increase of the numbers of primary and secondary dengue infections of 3.8% and 6.5%, respectively [42]. Another potential vector control strategy, based on the Release of Insects carrying a Dominant Lethal (RIDL) was studied by Atkinson *et al.* [47]. The RIDL male mosquitoes mate with wild females producing eggs dying prematurely before development to the adult stages [48]. As death can occur before or after the larval stage, in which density-dependent competition occurs [49], the authors analyzed both ‘early-lethal’(before larval stage) and ‘late-lethal’ (after larval stage) strategies combined with different policies for the release of RIDL males:

1. Proportional policy: number RIDL male mosquitoes kept in a fixed proportion to adult females,
2. Constant policy: maintaining the number of RIDL mosquitoes constant,
3. Trajectory policy: increasing the proportion of RIDL mosquitoes as the number of females decreases to maintain an exponential decline of infected female mosquitoes.

Atkinson *et al.* [47] derived the conditions on parameters governing vector-control for disease eradication. A larger number of genetically modified mosquitoes was found necessary with the constant policy, and the fastest eradication was obtained with the trajectory policy. Moreover, the ‘late-lethal’ strategy, for which death of the progeny occurs after the larval stage, would require a lower number of RIDL mosquitoes due to density-dependent competition in the larval stage [49]. The authors concluded that the RIDL strategy could be considered as a vector-control strategy in dengue-endemic areas.

#### Alternative transmission routes

Wei *et al.* developed a dengue transmission model including direct between-host transmission (which is strictly only expected through blood transfusion, bone marrow transplantation or needle sticks) and represented the extrinsic incubation period using a time delay [50], [51], [52]. Threshold conditions for the existence of an endemic equilibrium were derived. The authors showed, using the time-delay as bifurcation parameter, that this equilibrium might become unstable and periodic solutions exist. This modelling framework permitted to show the instability of the endemic equilibrium for relatively long extrinsic incubation periods. However, the authors assumed a direct transmission between infected and susceptible hosts to mimic the transmission through blood transfusion, transplantation or needle sticks. Although the risk of acquiring healthcare-related dengue exists in endemic areas, it remains a very rarely reported event and according to Wilder-Smith *et al.* dengue is not considered as a risk to blood safety [53].

Transovarial (or vertical) transmission of dengue virus in *Aedes aegypti* and *albopictus* is well documented under both experimental [54], [55], [56] and field conditions [57], [58], [59], [60] and could explain its persistence over inter-epidemic periods in endemic areas [60], [61]. Esteva and Vargas studied the impact of vertical transmission on dengue disease dynamics, assuming that a proportion of vector recruitment occurred in the infectious class [62]. This model also included a mechanical transmission after an interrupted meal by a mosquito on an infectious person. Conversely to mechanical transmission, which had a weak impact, vertical transmission was found to increase dramatically the endemic proportion of infectious vectors, which could favour the persistence of the virus in areas with low human densities [35], [63], [64]. Similar conclusions were drawn by Coutinho *et al.* from a model structure accounting for a pre-adult stage with periodic maturation rates and assuming that a proportion of the eggs laid by infected mosquitoes were vertically infected [65]. The authors identified transovarial transmission as a possible explanation of dengue overwintering and explained, using a time-dependent threshold condition [66], the delay observed between the peaks in vector density and in dengue cases. Adams *et al.* studied the influence of vertical transmission efficiency on the time to disease extinction, combining deterministic and stochastic approaches [67]. A pre-adult stage was also included along with a periodic recruitment rate and diapause period (persistence of eggs under unfavourable conditions *e.g.* winter/dry season) [68], [69]. The authors concluded that vertical transmission efficiency should exceed 20%–30% to significantly impact the transmission dynamics. Although such levels of efficiency were not obtained in experimental conditions, the authors pointed out the need to estimate the vertical transmission efficiency from field settings. Based on the framework developed by Coutinho *et al.* [65], Burattini *et al.* assumed a linear increase of egg-carrying capacity with time, representing the impact of environmental changes due to global warming and the increasing number of new constructions providing larger amount of breeding sites for mosquitoes [70]. Burattini *et al.* studied the impact of vector control policies that aimed at increasing the mortality rate of the adult mosquitoes and larvae by insecticide fogging and larvicide pulverization in breeding sites. These control strategies were tested separately or combined. Their results showed a better efficiency when implemented simultaneously and highlighted the importance of seasonality, which had a major influence both on the transmission process and on the potential efficacy of vector control policies. During the Singapore outbreak in 2003–2005, the vector control policy was based on a “search and destroy” strategy aiming at reducing the number of potential breeding sites. Model simulations permitted to reproduce visually/qualitatively well the Singapore data both before and after interventions for vector control [70].

#### Age-structured models and vaccination

In the 1960's, Dengue Hemorrhagic Fever was recognized as a childhood disease [71], [72], becoming a leading cause of child hospitalizations and deaths in southeastern Asia in the mid 1970's [10]. A retrospective seroepidemiological study of the 1981 dengue outbreak in Cuba reported 14.5 times more DHF/DSS deaths in children (under 15 years) than in adults [73]. Two age-structured models were developed dividing the host population into two broad age classes (children under 15 years and adults) [74], [75]. Pongsumpun *et al.* assumed a lower transmission rate in adults and conducted a stability analysis [74]. The model proposed by Supriatna *et al.* included supplementary disease stages representing symptomatic hosts, assumed to be isolated in hospital avoiding interaction with the vector [75]. This study focused on the potentially negative impact of vaccinating infectious children due to the presence of cross-reactive antibodies. Two potential negative impacts were assumed: (1) a longer infectious period; (2) an increase of virulence and likelihood of experiencing symptoms. The authors concluded that a longer infectious period would increase the effective reproduction number 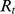, and vaccination would be counterproductive. Conversely, increasing the proportion of children showing symptoms, and subsequently removing them from the transmission process, was found to reduce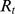.

Garba *et al.* also studied the impact of vaccination assuming that both the hosts and vectors were able to transmit the virus during their latent phases [76]. Based on the work of Shorami *et al.* [77], the author compared two model structures:

1. a standard (frequency-dependent) incidence possibly resulting in backward bifurcation: reducing 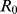 below 1 would not necessarily lead to disease control
2. a mass-action incidence with a constant host population, removing the backward bifurcation phenomenon.

Garba *et al.* concluded that vaccination would always have a positive effect with a decrease of the total number of infections [76]. However, the existence of four immunologically distant serotypes could lead to different conclusions due to possible reinfections in the presence of cross-reactive antibodies.

### Multi-serotype models

The dynamics of dengue infection is complex due to four co-circulating serotypes in many endemic areas, and the absence of long-term cross-immunity. The first large documented dengue outbreak occurred in Cuba in 1981 with more than 10000 severely affected persons and 158 deaths [78]. This outbreak followed a previous epidemic in 1977 which resulted in a seroprevalence level as high as 44.6% of the Cuban population (2.7% before 1977) [79]. The subsequent 1981 outbreak thus allowed to study the role of secondary infections as a potential risk factor for severe clinical disease [80]. Kouri *et al.* conducted a follow-up study of 124 children and 104 adults with severe clinical disease, 98% of whom exhibited a secondary serological response [78]. This and other studies on the risk factors for DHF/DSS in endemic areas support the hypothesis of Halstead regarding the importance of subsequent infections with different dengue serotypes inducing antibody-dependent enhancement (ADE) [23], [81], [82], [83], [84], [85].

Although the exact role of cross-reactive antibodies on dengue transmission is not fully understood, two main hypotheses regarding ADE were commonly adopted in modelling studies (Figure 3; Table 3):

1. Susceptibility enhancement: a first exposure to a serotype increases the susceptibility of infection with a second serotype.
2. Transmission enhancement: higher infectivity of individuals infected for the second time (secondary infected individuals).

**Table 3:** Formulations of antibody cross-reaction hypotheses in host-to-host transmission models.

Force of infection (FOI)	Range of Enhancement parameter	Type of enhancement	References	Susceptible Individuals exposed to the FOI
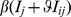	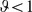	Reduced transmission	[99], [113], [114] *	Individuals susceptible to all serotypes or to serotype 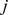 only
	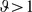	Transmission enhancement of secondary infected individuals	[99], [100], [102], [103], [104], [105], [106], [107], [108], [112]	
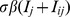	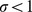	Cross-immunity between serotypes (also called “immunological distance”)	[105], [111], [112], [115]	Primary infected individual with serotype different from serotype 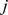
	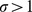	Susceptibility enhancement	[106], [112]	

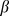
 is the transmission rate, 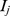 represents the number of individuals infected with serotype 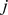 and 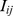 the number of individuals subsequently infected with serotypes 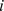 and 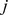.

* In references [113], [114], Aguiar *et al.* assumed that a proportion of secondary infected individuals contribute to a lesser extent to the epidemic process due to hospitalisation or isolation. This assumption is based on the evidence that secondary infections are more likely to produce severe clinical expression of the disease. As the antagonist relationship between previously acquired antibodies and secondary infection with an heterologous serotype is certainly involved in the intra-individual disease evolution, we classified this assumption as depending on the antibody cross-reaction hypotheses.

#### Vector-host transmission

Derouich and Boutayeb developed a model with two subsequent infections at separate time-intervals, considering that the first epidemic had ended when the second occurred [86]. Including vaccination in their model, the authors concluded that, in the absence of tetravalent vaccine, partial vaccination could be part of a control strategy. However, ADE could induce counterproductive effects.

Esteva and Vargas built a two-strain model on the basis of their single-serotype model [19], [87]. The vector population was subdivided into a susceptible class and two serotype-specific infectious classes For each serotype, the host population was governed by a SIR model. However, individuals who recovered from (a primary) infection with one serotype could be infected with the second one (secondary infection). A scaling factor (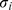) was applied to the force of infection representing the susceptibility enhancement due to ADE (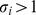) or cross-immunity (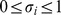) in people who recovered from primary infection. Threshold conditions for the coexistence of two strains, greatly favoured by susceptibility enhancement, were established. Feng *et al.* used the same approach to represent ADE and cross-immunity [88]. However, their model did not have an explicit state for individuals who recovered from primary infections. Thus, the duration of the infectious period in solely primary infected individuals was dependent on the time to secondary infection, leading to overestimations of this duration and the number of infectious individuals. Feng *et al.* demonstrated the existence of an unstable endemic equilibrium, and the general result was the competitive exclusion of one strain, due to selective pressure exerted by ADE.

The majority of dengue infections is asymptomatic or induces only mild symptoms (DF). However, since only severe cases (DHF/DSS) are reported, the actual incidence is likely underestimated. Nuraini *et al.* added a supplementary compartment to Esteva's model accounting for severe DHF following a secondary infection, and assumed that a fixed proportion 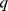 of secondary infected individuals developed clinical DHF [87], [89]. Severely affected individuals were assumed not to take part in the transmission process since their hospitalization was assumed to rule out interaction with the vector. Sriprom *et al.* also accounted for symptomatic and asymptomatic compartments but assumed that asymptomatic individuals were not able to transmit the virus to susceptible mosquitoes because of low viral load [90]. The mathematical analyses of these two models, studying equilibria stability and threshold values, were similar to Esteva's [87], [89], [90].

Bartley *et al.* [91] proposed a more complex modelling structure, representing the evolution of the immune response:

1. Short-term (2 to 9 months) and partial cross-immunity.
2. Sub-neutralizing antibody-level inducing an increase in the infectivity in the secondary infected host and, consequently, impacting the transmission rate from host to vector.
3. Immunity to one serotype without cross-reaction with other serotypes.

Cross-reaction between serotype-specific antibodies and heterologous virus serotypes induces a higher viral replication in both *in vitro* and *in vivo* conditions [85], [92]. This enhanced replication, pointed out as a major risk factor for DHF/DSS, could also have an effect on the transmission process. Bartley *et al.* [91] introduced a scaling factor (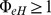) on the force of infection exerted by secondary infected hosts with sub-neutralizing antibody level on the vector population. The vector population was governed by a Susceptible-Exposed-Infectious (SEI) model, assuming 50% of infected vectors for each serotype. The most important feature in this model relied on the inclusion of seasonality in parameters (recruitment, mortality and biting rates, duration of EIP) estimated from specific entomological studies in Bangkok [93], [94], [95], [96]. Univariate sensitivity analysis on each parameter was performed to study its impact on the transmission process in the absence of seasonality. The duration of the infectious period in the host as well as the biting and vector mortality rates were highlighted as essential parameters. Model outputs were compared with epidemiological data in Thailand leading to the conclusion that the main determinants of seasonality were incorporated. Wearing and Rohani developed a four-serotype model on the basis of the work of Bartley *et al.* [97]. ADE was represented as increasing the susceptibility in primary infected hosts with a possible temporary ADE due to the decrease of cross-reactive antibodies below enhancing levels. A periodic recruitment rate of the vector population and variations in serotype virulence (assumed to increase disease induced mortality) were also included in Wearing's model. Using this complex representation, the authors concluded that seasonality was necessary to explain intra-annual dynamics and temporary cross-immunity was sufficient to obtain inter-epidemic periods of three years observed in dengue endemic areas.

The impact of vector control strategies was rarely investigated in a multistrain framework. Recently, Alphey *et al.* proposed a two-serotype model to evaluate the impact of RIDL on dengue transmission [98]. Using a formulation close to Atkinson *et al.* [47], the authors considered a constant ratio between RIDL male and wild female mosquitoes and established the condition on this proportion for disease eradication. Although accounting for susceptibility enhancement to reproduce inter-epidemic periods, the authors ignored the seasonality factor, which was shown essential to explain intra-annual variations in dengue incidence [97].

#### Host-to-Host transmission

In contrast with previous studies, modelling explicitly the vector population, Ferguson *et al.* developed a two-serotype model and assumed the time scale for transmission sufficiently short and the mosquito population sufficiently dense to consider direct transmission between two host subpopulations [99], [100]. This model was not specifically designed to study the dengue transmission process but aimed at understanding the cross-reactive effect of antibodies generated by a primary infection on a secondary infection. This antibody-dependent effect, observed for a wide variety of viruses [101], was represented as decreasing (partial cross-immunity) or increasing (ADE) the transmission by secondary infected individuals. Numerical analysis permitted to demonstrate oscillatory chaotic behaviour with large inter-epidemic periods of several years and easy coexistence of strains. Billings *et al.* further incorporated two vaccination schemes in Ferguson's model: (i) a single serotype vaccine and (ii) vaccination against both strains assuming that one host can only be vaccinated against one strain [102]. The authors derived the threshold conditions for coexistence, eradication of one strain (strategy (i)), and eradication of both strains (strategy (ii)). However, Billings *et al.* concluded that, with standard dengue parameters (Table 4), the eradication of both strains using separate serotype vaccines would not be feasible.

**Table 4:** Dengue model parameters in host-to-host transmission approaches.

Parameter	Definition	Value
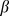 (year^−1^)	transmission rate	200–400
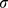 (year^−1^)	duration of the infectious period in hosts	100
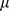 (year^−1^)	1/host lifespan	50
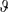	ADE factor	1–5

With these parameter values, the basic reproduction number range is 2–4.

ADE: antibody-dependent enhancement. Here, with values greater than 1, the secondary infected individuals are assumed to contribute to a greater extent than primary infected individuals to the transmission process (Table 3).

Mathematical models were developed to assess the impact of co-circulation of the four serotypes on the course of infection. Most of these approaches were based on the work of Ferguson *et al.* [99] and generalized to more than two serotypes [100], [103], [104], [105], [106], [107], [108]. Although there is no evidence of complete immunity to all serotypes after two subsequent infections, third and fourth infections are rarely reported since they have no or only minor consequences for the clinical disease outcomes [9].

All but one [107] of these models assumed individuals to be immune to all serotypes after two sequential infections, thereby drastically reducing the number of equations [100], [103]. Chronologically, Cummings *et al.* showed that the solutions of their model exhibited a wide range of behaviours from a stable fixed point, for a low level of enhancement, to desynchronized chaotic behaviour [103]. High increases of transmission due to ADE were found to induce large amplitude oscillations exhausting the pool of susceptibles and thus eliminating the fitness advantage of ADE. Schwartz *et al.* introduced seasonal transmission rates using a sinusoidal function [104]. Intuitively, this periodic forcing would be expected to break the desynchronization obtained by Cummings *et al.* [103]. However, the results obtained by Schwartz showed that the periodicity in transmission rates was not sufficient to cause synchronization between serotypes [104]. Billings *et al.* investigated the impact of vaccination against a single serotype and showed the negative effect of such a vaccination strategy [100]. Bianco *et al.* studied the interplay between cross-immunity and ADE showing that weak cross-immunity would lead to a stable endemic steady state whereas strong cross-immunity favoured chaotic outbreaks [105]. This model was further developed to study the impact of migration between two distinct populations in the presence of multiple circulating strains. The inclusion of migration between the two population-patches resulted in the stabilization of the system, especially when the asymmetric transmission rates in the respective patches were considered [108]. Recker *et al.* decomposed ADE into two different mechanisms: (i) susceptibility-enhancement to secondary infection after a primary infection and (ii) transmission enhancement in secondary infected hosts [106]. This decomposition permitted to produce dynamic behaviour showing asynchrony between serotypes and inter-epidemic periods (3 to 5 years) in accordance with outbreak data [109]. Moreover, model outputs showed a good qualitative agreement with dengue data from 1973 to 1999 in Thailand both for serotype dynamics and disease incidence. Lourenço and Recker expanded this model by including a vector component, and studied the introduction of a novel virus genotype into a four-serotype endemic population [110]. Wikramaratna *et al.* compared the dynamic behaviour of two models assuming (i) complete immunity after two subsequent infections and (ii) the inclusion of tertiary and quaternary infections [107]. Although these assumptions did not modify the global behaviour, the force of infection increased when accounting for tertiary and quaternary infections, decreasing significantly the age at first infection.

Kawagushi *et al.* [111] also studied the impact of cross-immunity, reflecting the “immunological distance between two different serotypes”, and ADE on the coexistence of the strains using an SIR formulation with two interacting populations, direct host-to-host transmission and possible simultaneous co-infection with the two serotypes. Secondary dengue infection being a major risk factor for DHF/DSS, the authors considered a mortality increase in secondary infected hosts. The analysis focused on the stability of the marginal (single-strain endemic) and the endemic two-strain equilibria. Assuming in a first step that only one strain (called “resident strain”) was endemic in the population, the impact of the introduction of a second strain was assessed. Mortality enhancement was found to generate a need for large immunological distance for strains to coexist. Adams and Boots used a similar framework to study the interaction between ADE and cross-immunity [112]. Ferguson's [99] and Kawagushi's [111] assumptions were combined in a single model: cross-immunity, transmission and mortality enhancement. Moreover, the authors included a susceptibility enhancement, increasing the force of infection exerted on primary infected hosts. The relative effect of each form of enhancement was tested in combination with cross-immunity confirming the results obtained in the previous studies. The authors performed further numerical simulations (not detailed in the article) showing that susceptibility and transmission enhancements had a cumulative impact “allowing coexistence of increasingly similar strains”. The effect of increased mortality, combined with the two other forms of enhancement, was weak and did not greatly influence the previous results.

Infection with one dengue serotype provides life-long immunity to that specific serotype but also a short-term cross-protection against infection with heterologous serotypes. Although different models accounted for immune cross-reaction leading to a decrease of the force of infection exerted on primary infected individuals [106], [111], [112], the influence of temporary cross-immunity on infection dynamics was rarely explicitly modelled [97]. Aguiar *et al.* developed a two-serotype model accounting for a period of temporary cross-protection after which primary infected individuals were considered as fully susceptible to infection with the alternate serotype [113]. Apart from the inclusion of cross protection, one original assumption in this model (only considered in two vector-host transmission models [75], [89]), stipulates that a proportion of secondary infected individuals could participate to a lesser extent to the force of infection due to hospitalization [113]. Whereas most direct host-to-host models considered increased transmission after a second infection, based on the fact that the viral load is higher in such cases, Aguiar *et al.* made the opposite assumption: “*the inverse ADE*”. Using numerical continuation methods for bifurcation analysis, the authors showed that the system exhibits deterministic chaotic behaviour in an unexpected parameter range only through inclusion of cross-immunity in previously existing models. Recently, this model was further extended by including a periodic transmission rate and importation of infective individuals in the population [114]. The analysis of the periodically forced model was close to the non-seasonal model. Seasonality was found necessary to reproduce intra-annual fluctuation. Moreover, combination of seasonality and importation of infectives permitted to reproduce qualitatively DHF incidence data in the Province of Chang-Mai in Thailand.

Nagao and Koelle suggested another possible additional benefit provided by cross-immunity called ‘clinical cross-protection’ [115]. During the period of cross-protection following primary infection, hosts could be infected by a heterologous strain without developing symptoms and would consequently acquire immunity to the challenging serotype [116]. The authors showed that the reduction of the force of infection could be counterproductive, because of a lower proportion of individuals acquiring multi-serotype immunity through ‘clinical cross-protection’, leading to a higher number of clinical manifestations. These results were supported by Chikaki and Ishikawa, who developed an age-structured model including a periodic vector population and differential transmission rates between serotypes [117]. Infections occurring during the clinical cross-protection period were assumed asymptomatic. These asymptomatic individuals contributed to a lesser extent to the transmission process. The authors concluded that the clinical cross-protection assumption, called in this study “unnatural transmission”; modified the dynamics of infection and could explain observations in Thailand, where large dengue outbreaks occur irregularly every few years.

## Discussion

Dengue is the major arbovirose (arthropod-borne virus) in the world and a leading cause of hospitalization and death among children in Asia [13], [72], [118]. It is especially prevalent in tropical regions, where the primary vector *Aedes aegypti* thrives. Although *Aedes albopictus* was shown to be less efficient for dengue transmission than *Aedes aegypti*, its role was clearly established in a few dengue outbreaks in areas free from the primary vector (*e.g.* in Japan in 1942 and more recently in Hawaii (2001)) [8]. However, the global expansion of this secondary vector, combined with the possible arboviral adaptation to alternative mosquito species, could give rise to dengue outbreaks in areas, which had been unaffected up till now [8], [119]. Dengue is a complex disease involving vector ecology, host immunity and other external factors. Recently, Banu *et al.* made a review on the impact of climate change and socio-environmental factors on dengue transmission, concluding that global warming could influence dengue epidemiology in the near future [120].

Fourty-two deterministic mathematical models were included in the present review, of which 18 single-serotype models were based on the basic framework proposed by Bailey [18]. These models differed in their formulations through the representations of the host and/or vector populations and permitted to analyze the possible transmission routes (direct and transovarial transmission) and control strategies (vector control, vaccination). The remaining 24 studies described multi-serotype models, mainly focused on the ADE phenomenon with different formulations of ADE consequences on dengue transmission (susceptibility, transmission and/or mortality enhancement). Although some of the selected articles accounted for variability by introducing stochastic perturbation in state variables [100], [103], in parameters [112] or through the development of the stochastic counterpart of the deterministic models [67], [97], [99], stochastic models were not the focus of this review. Focks *et al.* developed a simulation model (Dengue Simulation Model: DENSiM) to study the spread of dengue in an urban context [121]. Entomological parameters were estimated from a stochastic weather-driven model of the *Aedes* mosquito population (Container-Inhibiting Mosquito Simulation Model: CIMSiM) [122]. This pair of stochastic models was used to study the transmission thresholds in terms of pupae per person [123]. Otero *et al.* developed stochastic models representing the evolution and spatial dynamics of the *Aedes aegypti* population in Buenos Aires [124], [125]. The resulting model, coupled with an epidemiological dengue model, showed that the timing of virus introduction within a population could have a huge impact on the final size of the epidemics [126]. The model was further improved through the inclusion of human mobility described in terms of complex networks [127]. Massad *et al.* also used the complex networks approach to analyze the geographical spread of dengue during the 2005 outbreak in Singapore [128]. Spatial heterogeneity was included in an individual based model by Favier et al. considering household structure for both the host and vector populations and host movements between households [129]. Other stochastic approaches were based on cellular automata models, highlighting the importance of seasonality and host mobility [130], [131], [132], [133], [134]. The analysis of hospitalization data from 72 provinces in Thailand revealed a radial geographic spread of the disease from the region of Bangkok [135]. Deterministic reaction-diffusion equations were also used to study the spatial dynamics of dengue [136], [137].

Another practical use of mathematical models focuses on the estimation of 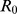 from field data [17]. Koopman *et al.* estimated 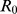 from the final sizes of epidemics in 70 Mexican localities with a mean value of 1.3 [138]. Ferguson *et al.* used a maximum likelihood method to analyze a sero-epidemiological survey, accounting for age- and serotype-specific sero-prevalence, which resulted in an estimated range of 1.38–8.47 [139]. Marques *et al.* evaluated 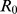 from the initial (exponential) growth rates of dengue epidemics in Brazil [140]. This method was further improved to analyze different dengue outbreaks [141], [142]. Massad *et al.* assessed the risk of yellow fever and chikungunya infection in dengue endemic areas [143], [144]. In doing so, they derived the basic reproduction number for yellow fever and chikungunya using epidemiological parameters and 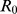 for dengue, which was estimated using either the final size or the intrinsic growth rate method. This permitted to evaluate the density of vectors per host in order to simulate chikungunya spread and the risk it presents to locals and travellers using the model framework described by Coutinho *et al.* [65], [66]. Chowell *et al.* [145] studied the impact of realistic distributions for the extrinsic and intrinsic (gamma-distributed) incubation periods on estimates of 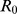 from the initial phase of the dengue epidemic curve. The authors concluded that the classical exponential distributions assumption leads to an overestimate of the basic reproduction number. Pinho *et al.* [146] used the framework proposed by Yang *et al.* [39] (accounting for pre-adult and adult vector stages) to estimate the basic and effective reproduction numbers from dengue outbreak data in Salvador, Brasil. Hsieh and Chen analyzed a two-wave dengue epidemic in Taïwan in 2007 using a multi-phase Richards model [147]. Supriatna et al. derived 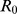 estimates from the mean age at infection using data from the 2002–2007 dengue outbreaks in Indonesia [148]. We refer to Johansson *et al.* for a review on 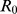 estimations for dengue [16]. Although all of these studies provide essential general insights on the potential for disease spread and impact of interventions, they were excluded from our selection process since we focused exclusively on the structural approaches used for dengue modelling. The differences between these structures are pivotal to understand projections of the impact of interventions on the transmission dynamics over time. Understanding the differences between different model structures and assumptions is therefore essential to further improve dengue models, and to test the plausibility of unknown transmission properties of the serotypes in relation to each other, as well as in relation to host and vector behavioural characteristics. Further specific data collections and providing access to such data for model-based research, would prove helpful to further advance this field, both in terms of developing and validating model structures and diseases hypotheses (*e.g.* ADE), and in terms of projecting the risks and benefits of prevention and control strategies, such as vaccination.

## Conclusion

In the present review, deterministic mathematical models for dengue infection were described and two main approaches were highlighted: 28 accounted for the vector population and 14 articles considered direct host-to-host transmission, most of which were based on the work of Ferguson *et al.* (eight articles; Figure 3) assuming transmission enhancement in secondary infected individuals [99]. However, as pointed out by Wearing *et al.*, transmission enhancement would impact the probability of infection (up to 1) for a susceptible mosquito when biting an infectious host [97]. Seasonality, reflecting the favourable and unfavourable conditions for the vector, was found to be essential to explain the intra-annual fluctuations in dengue cases. Another assumption related with antibody cross-reaction relies on the susceptibility enhancement in primary infected individuals. Although immunity to heteorologous serotypes was clearly identified as a major risk factor for severe clinical expression after a secondary infection, to our knowledge, there is no evidence for an overall increase in susceptibility to a second dengue virus. However, the antibody-dependent enhancement phenomenon is an intra-host process leading to a higher peak of viremia in multiple infected individuals which could, in turn, increase the transmission rate from hosts to vectors (and thus support transmission enhancement) [1], [72].

Dengue clinical manifestations range from asymptomatic or atypical flu-like symptoms to severe expressions (Dengue Hemorrhagic Fever (DHF) or Dengue Shock Syndrome (DSS)). Three multiserotype models distinguished explicitly symptomatic from asymptomatic stages [89], [90], [113]. Nuraini *et al.* included a “severe DHF component” in their model and considered that symptomatic individuals were not involved in the transmission process because of hospitalization [89]. Aguiar *et al.* assumed that a hospital admission would decrease the transmission rate of severely affected symptomatic individuals [113]. However, these two assumptions are doubtful because the viral load peaks prior to hospitalization and, as stated by Kuno, the presymptomatic viremia period could be an important factor in the transmission process [35]. In contrast with these two approaches, Sriprom *et al.* made the opposite assumption considering that the virus can only be transmitted to the vector by symptomatic individuals (which they defined as having DHF), due to low viral loads in asymptomatic persons [90]. However, the counterexample of the DF epidemics in Cuba in 1977, with more than 0.5 million infections [78], clearly showed the transmission potential of individuals presenting comparatively milder symptoms. Therefore, it is important in models distinguishing asymptomatic from symptomatic patients to identify the proportion of each group, and their role in the transmission process, whilst using biologically plausible transmission parameters.

Newton and Reiter [21] assumed a differential biting rate in susceptible and infectious mosquitoes based on studies on other vector-borne diseases [149], [150]. This assumption was relaxed in most of the other studies following the conclusions of Putnam *et al.* [44]. However, a recent study showed an increase of locomotor activity in dengue infected mosquitoes, supporting the results of Platt *et al.* [43] and the assumption of Newton and Reiter [45].

For these reasons, even if host-to-host models reproduced qualitatively the main features of dengue epidemics, the representation of the vector population could be pivotal when modelling dengue to understand the relationship between vector abundance, external factors (*eg.* temperature, rainfall) and dengue incidence. However, due to the number of maturation stages from egg to adult, the representation of the vector population should be chosen with care and parsimony to avoid unnecessary uncertainties in model parameters.

In the absence of a tetravalent vaccine, the only effective preventive measures are based on vector control strategies, which can be assessed through mathematical models [39], [40], [70]. According to Burattini *et al.* [70], the combination of different control measures (pulse larvicide, insecticide and removal of breeding sites) was found the most effective strategy and permitted to reproduce qualitatively well the outcome of the intervention carried out during the 2005 outbreak in Singapore. Moreover, they showed that the inclusion of seasonality influenced drastically the impact of vector control. This observation is in line with the work of Yang and Ferreira, who identified the optimal period for each control strategy [39]. More recently, Luz *et al.* accounted for insecticide resistance in mosquitoes and performed an economic assessment of vector control strategies based on insecticide application [40]. Although larvicide application was found to reduce dramatically the vector population in the short term, evolution of resistance could produce counterproductive effects over time. The economic evaluation of different combinations of control strategies permitted to identify the use of multiple adulticide applications as the most cost effective intervention. The inclusion of mechanical control (removal of breeding sites) could however modify this result since it would affect both non-resistant and resistant vector population. Two studies considered the use of RIDL (Release of Insects carrying Dominant Lethal) strategy to control the vector population and concluded that this strategy could enable disease eradication in dengue endemic areas [47], [98]. Moreover, RIDL strategy was found more effective than vector control based on insecticide use [98]. Developing dengue vaccines is challenging for multiple reasons [151]. First, the antibody-dependent enhancement requires the vaccine to combine all four-serotype antigens to avoid adverse effects. Second, vaccine induced immunity should not wane below protective levels for any serotype. Finally, dengue vaccination should be cost-effective to be financially sustainable in low and middle-income countries. Several tetravalent dengue vaccine candidates have shown promise in clinical trials [152].

Ferguson *et al.* estimated from an age-stratified sero-epidemiological survey that 85% of the birth cohort should be vaccinated to achieve elimination, which can be challenging in many countries [139]. Furthermore, this represents an underestimate since the vaccine was assumed to provide complete protection against the four dengue serotypes. Among the 42 selected models, five included vaccination strategies (two single- and three multi-serotype models; Figure 3) [75], [76], [86], [100], [102]. However, only one considered four serotypes [100], while exploring the (adverse) impact of single serotype vaccination at 100% efficacy. Even with a “perfect” four-serotype, affordable and cost-effective vaccine, such a level of efficacy is unlikely. Vaccine failures could increase the risk of severe clinical cases through ADE. In such a case, a combination of vaccination and vector-control would be necessary for disease eradication. Moreover, decreasing the vector density through vector control reduces the basic reproduction number, which is proportional to the vector-host ratio. Clearly, such reduction lowers the vaccination coverage eradication threshold. Therefore vector control and vaccination should be combined, especially during the first years after vaccine introduction when vaccination coverage may not be sufficiently high to achieve herd immunity. Although the relationship between immunity, pathology and disease dynamics is fairly well known, large uncertainties persist about the immuno-epidemiological mechanisms acting on dengue transmission (transmission or susceptibility enhancement, role of short-term cross-protection). As shown in Table 3, most modelling frameworks assumed that antibody-dependent enhancement would increase the transmission rate in secondary infected individuals. Infection with a specific serotype would induce permanent immunity against that serotype and potentially also short-term cross-protection against heterologous serotypes, thus modifying the transmission dynamics and disease progression in infected people [91], [97], [105], [108], [113], [114], [115], [117]. In order to study the potential impact of widespread dengue vaccination on the disease burden, it would therefore be essential to understand the differences between naturally-acquired and vaccine-induced immunity. The WHO-VMI Dengue Vaccine Modeling Group presented a set of ten questions regarding the possible interactions of vaccine-induced immunity and dengue dynamics and/or pathology [153]. Our review of deterministic model structures is timely with respect to these questions. Perhaps our main finding in this respect is that the inclusion of the vector component in a four-serotype model would be necessary to identify the best combination of vector-control and vaccination strategies in dengue endemic areas.
